# From Alpha to Delta—Genetic Epidemiology of SARS-CoV-2 (hCoV-19) in Southern Poland

**DOI:** 10.3390/pathogens11070780

**Published:** 2022-07-08

**Authors:** Emilia Morawiec, Maria Miklasińska-Majdanik, Jolanta Bratosiewicz-Wąsik, Robert D. Wojtyczka, Denis Swolana, Ireneusz Stolarek, Michał Czerwiński, Aleksandra Skubis-Sikora, Magdalena Samul, Agnieszka Polak, Celina Kruszniewska-Rajs, Adam Pudełko, Marek Figlerowicz, Anna Bednarska-Czerwińska, Tomasz J. Wąsik

**Affiliations:** 1 Department of Microbiology, Faculty of Medicine in Zabrze, Academy of Silesia in Katowice, 41-800 Zabrze, Poland; e.morawiec@gyncentrum.pl; 2 Gyncentrum, Laboratory of Molecular Biology and Virology, 40-851 Katowice, Poland; michalczerwinski1992@gmail.com (M.C.); a.skubis-sikora@gyncentrum.pl (A.S.-S.); m.samul@gyncentrum.pl (M.S.); a.polak@gyncentrum.pl (A.P.); c.kruszniewska-rajs@gyncentrum.pl (C.K.-R.); a.pudelko@gyncentrum.pl (A.P.); czerwinskaa002@gmail.com (A.B.-C.); 3 Department of Histology, Cytophysiology and Embryology, Faculty of Medicine in Zabrze, Academy of Silesia in Katowice, 41-800 Zabrze, Poland; 4 Department of Microbiology and Virology, Faculty of Pharmaceutical Sciences in Sosnowiec, Medical University of Silesia in Katowice, 41-200 Sosnowiec, Poland; mmiklasinska@sum.edu.pl (M.M.-M.); rwojtyczka@sum.edu.pl (R.D.W.); dswolana@sum.edu.pl (D.S.); 5 Department of Biopharmacy, Faculty of Pharmaceutical Sciences in Sosnowiec, Medical University of Silesia in Katowice, 41-200 Sosnowiec, Poland; jbrat@sum.edu.pl; 6 Department of Molecular and Systems Biology, Institute of Bioorganic Chemistry Polish Academy of Sciences, 61-704 Poznań, Poland; istolarek@ibch.poznzn.pl (I.S.); marek.figlerowicz@ibch.poznzn.pl (M.F.); 7 American Medical Clinic, 40-851 Katowice, Poland; 8 Department of Cytophysiology, Chair of Histology and Embryology, Faculty of Medical Sciences in Katowice, Medical University of Silesia in Katowice, 40-055 Katowice, Poland; 9 Department of Molecular Biology, Faculty of Pharmaceutical Sciences in Sosnowiec, Medical University of Silesia in Katowice, 41-200 Sosnowiec, Poland; 10 Department of Clinical Chemistry and Laboratory Diagnostics, Faculty of Pharmaceutical Sciences in Sosnowiec, Medical University of Silesia in Katowice, 41-200 Sosnowiec, Poland; 11 Department of Gynecology and Obstetrics, Faculty of Medicine in Zabrze, Academy of Silesia in Katowice, 41-800 Zabrze, Poland

**Keywords:** SARS-CoV-2 variants, molecular epidemiology, phylogenetics, spike mutations, viral genomics, whole-genome sequencing, Poland

## Abstract

In Poland, the first case of SARS-CoV-2 infection was confirmed in March 2020. Since then, many circulating virus lineages fueled rapid pandemic waves which inflicted a severe burden on the Polish healthcare system. Some of these lineages were associated with increased transmissibility and immune escape. Mutations in the viral spike protein, which is responsible for host cell recognition and serves as the primary target for neutralizing antibodies, are of particular importance. We investigated the molecular epidemiology of the SARS-CoV-2 clades circulating in Southern Poland from February 2021 to August 2021. The 921 whole-genome sequences were used for variant identification, spike mutation, and phylogenetic analyses. The Pango B.1.1.7 was the dominant variant (n = 730, 89.68%) from March 2021 to July 2021. In July 2021, the B.1.1.7 was displaced by the B.1.617.2 lineage with 66.66% in July 2021 and 92.3% in August 2021 frequencies, respectively. Moreover, our results were compared with the sequencing available on the GISAID platform for other regions of Poland, the Czech Republic, and Slovakia. The analysis showed that the dominant variant in the analyzed period was B.1.1.7 in all countries and Southern Poland (Silesia). Interestingly, B.1.1.7 was replaced by B.1.617.2 earlier in Southern Poland than in the rest of the country. Moreover, in the Czech Republic and Slovakia, AY lineages were predominant at that time, contrary to the Silesia region.

## 1. Introduction

The genetic diversity of SARS-CoV-2 (hCoV-19), together with the development of next-generation sequencing (NGS) methods, offers an opportunity to track the expansion of the COVID-19 epidemic across different populations. The ongoing pandemic impelled the international research community to generate a large amount of viral genome sequences stored in the Global Initiative on Sharing All Influenza Data (GISAID) database which permits monitoring virus molecular evolution in virtually real time. Despite SARS-CoV-2’s relatively low mutation rate, estimated at 0.8–2.38 × 10^–3^ nucleotide substitutions per site per year, it accumulates genetic changes over time, as evidenced by newly emerging variants [1]. The accumulation of acquired fixed mutations in a viral genome permits phylogenetic analyses and gives insights into the origins and routes of SARS-CoV-2’s spread, while tracking of low-frequency mutations and their divergence over time within infected individuals can provide insights into the dynamics of intrahost evolution, thus allowing the connection of particular variants with different transmissibility, immune escape, or pathogenesis patterns [2,3]. The majority of newly emerging variants have little to no impact on the virus properties, without any influence on the overall epidemiological situation. However, some mutations, mainly within gene-*S*-encoding spike protein, significantly affect the virus properties, such as how easily it spreads, the disease severity, the effectiveness of vaccines, chemotherapeutics sensitivity and specificity of diagnostic tests, or other public health and social measures [4]. These variants are classified as variants of concern (VOC). The established nomenclature systems for naming and tracking SARS-CoV-2 genetic lineages by GISAID, Nextstrain, and Pango are currently in use [4,5,6].

In September 2020 in the UK, the first VOC belonging to the B.1.1.7 lineage and harboring 17 characteristic mutations, eight of which were within gene *S,* was detected. The new lineage carried N501Y substitution in the receptor-binding domain (RBD), the HV 69–70 deletion (Δ), and the P681H substitution in the furin cleavage site which resulted in increased virus transmissibility and virulence. In December 2020, the B.1.1.7 variant accounted for 25% of sequenced genomes and by January 2021 was responsible for more than 70% of new infections in the UK [7]. Variant B.1.1.7 was introduced to Poland by citizens working in the UK returning to the country in the last 2 weeks of December 2020. Spreading rapidly, it became the dominating variant in Poland by the third week of February 2021. Its supremacy has been ended by the Pango B.1.617.2 variant. It was first detected in India in late 2020. This VOC harbors substitutions T478K, P681R, and L452R, which are known to increase transmissibility, severity, and immune escape [8,9]. In Poland, it was first detected on 26 April 2021 and it became dominant by the second week of June 2021, as was noted in our study.

The aim of our study was to present the molecular epidemiology of the SARS-CoV-2 lineages circulating in Southern Poland between February 2021 and 8th August 2021. Based on whole-genome sequencing, variant identification, analysis of spike protein mutation, and phylogenetic analysis of 814 viral genomes, we demonstrate the variants’ evolution. Our data collected in Southern Poland were also compared with the sequences from Poland as well as from the Czech Republic and Slovakia, countries bordering the analyzed region.

## 2. Results

### 2.1. The Prevalence of SARS-CoV-2 Variants (hCoV-19) in Southern Poland

**Figure 1:**
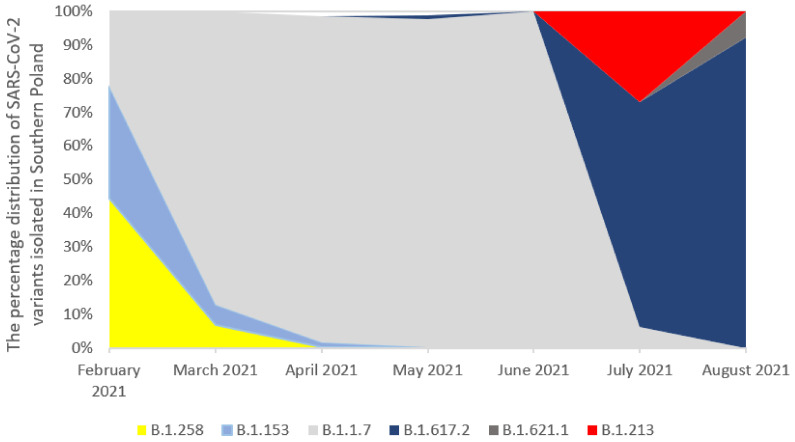
The percentage distribution of SARS-CoV-2 variants isolated in Southern Poland from February 2021 to 8th August 2021 based on whole-genome sequencing. The Pango lineage naming system for variants was used. In February 2021, the B.1.258 virus variant was dominating (44.4%), followed by the B.1.153 (33.33%). In March 2021, the B.1.160 variant was replaced by the B.1.1.7 which completely dominated lineages circulating in Poland (87.17%) and lasted for the next three months (96.96%, 97.37%, and 100%, respectively). In May, lineage B.1.617.2 appeared for the first time, which accounted for 1.27%. In July 2021, the B.1.1.7 variant was completely displaced by the B.1.617.2 lineage, which occurred with 66.66% frequency. Moreover, another peak of variant B.1.213 (28.12%) was recorded in July 2021. In August 2021, the most common virus type was the B.1.617.2—92.3%. The analysis was carried out in the Nextstrain domain.

From the 921 sequenced samples, 107 were rejected due to the large number of missing nucleotides in sequences (>1500). The remaining 814 samples were classified into variants and analyzed for mutations in the spike protein. The B.1.1.7 was the dominant variant among the sequenced samples (n = 730, 89.68%). The B.1.258 was noted in 15 sequences (1.84%), while the B.1.213 variant was observed in 8 sequences (0.98%). The B.1.153 was identified in 14 samples (1.72%). The B.1.160 variant appeared in the period from February to March 2021; then, it was replaced by the B.1.1.7 one. Interestingly, the next peak of the B.1.160 variant occurred in July 2021, but it was the B.1.213 lineage. In July 2021, variant B.1.617.2 dominated the studied population and replaced variant B.1.1.7. Thirty-nine (4.79%) sequences were identified as B.1.617.2. The analysis of the above data suggests that the diversity of variants circulating in the studied population is not large. We observed the domination of B.1.1.7 and B.1.617.2 variants over time. The percentage distribution of the main SARS-CoV-2 variants isolated in Southern Poland from February 2021 to August 2021 is presented in Figure 1.

### 2.2. The Prevalence of SARS-CoV-2 Main Variants (hCoV-19) in Poland, the Czech Republic, and Slovakia

**Figure 2:**
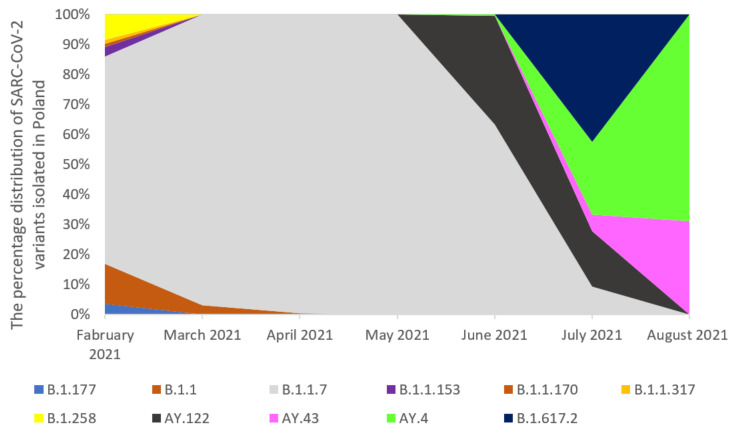
The percentage distribution of SARS-CoV-2 variants isolated in Poland (except our sequences shown in Figure 1) from February 2021 to 8th August 2021 based on GISAID. In February, March, April, May, and June 2021, the B.1.1.7 virus variant was dominating (57.56%, 90.14%, 98%, 96%, and 92%, respectively). In July 2021, the B.1.1.7 variant was completely displaced by the B.1.617.2 lineage, which occurred with 41% frequency. Moreover, another peak of variant B.1.213 (28.12%) was recorded in July 2021. In July 2021, the AY.122, (17.79%), AY.43 (5.41%), and AY.4 (23.4%) variants appeared.

**Figure 3:**
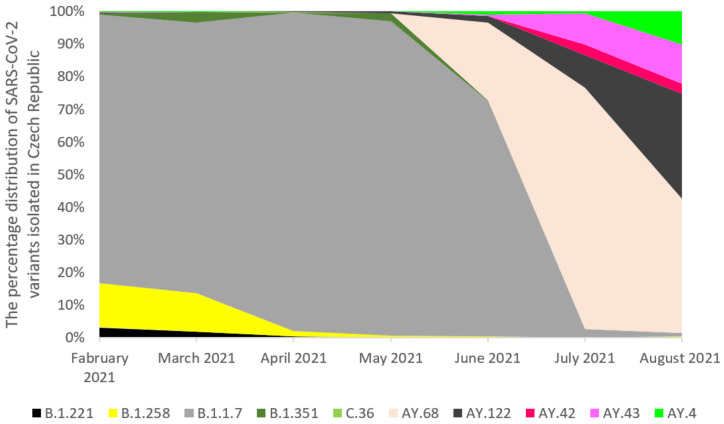
The percentage distribution of SARS-CoV-2 variants isolated in the Czech Republic from February 2021 to 8th August 2021 based on whole-genome sequencing. In February 2021, the B.1.1.7 virus variant was dominating (79%), followed by the B.1.258 (13%). Variant B.1.1.7 dominated over the next three months with a frequency: 81%, 92.2% and, 86.11, respectively. In June 2021, there were also first cases of variants AY.68 (21%) and B.1.617.2 (5.37%), but the B.1.1.7 variant was still dominating (64%). In July and August 2021, the AY.68 virus variant was dominating (53.58% and 25.4%), followed by the AY.122 (7.22% and 19.9%).

**Figure 4:**
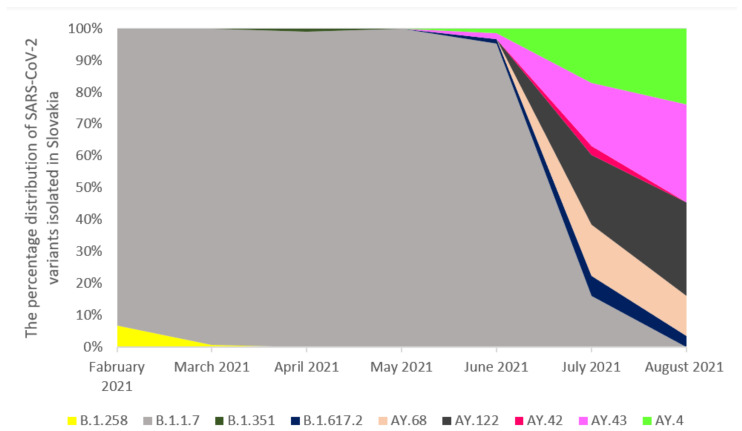
The percentage distribution of SARS-CoV-2 variants isolated in Slovakia from February 2021 to 8th August 2021 based on whole-genome sequencing. The PANGO lineage naming system for variants was used. From February 2021to June 2021, the B.1.1.7 virus variant was dominating (69%, 90%, 92%, and 98%, respectively). In July 2021, the B.1.1.7 variant was slowly replaced by AY.122 (15.62%), AY.43 (14.13%), and AY.4 (12.13%) which were dominating lineages circulating in Slovakia (87.17%). In August 2021, the B.1.1.7 variant was completely displaced by the AY.122 (21%), AY.4 (17%), and AY.43 (22%) lineages.

**Figure 5:**
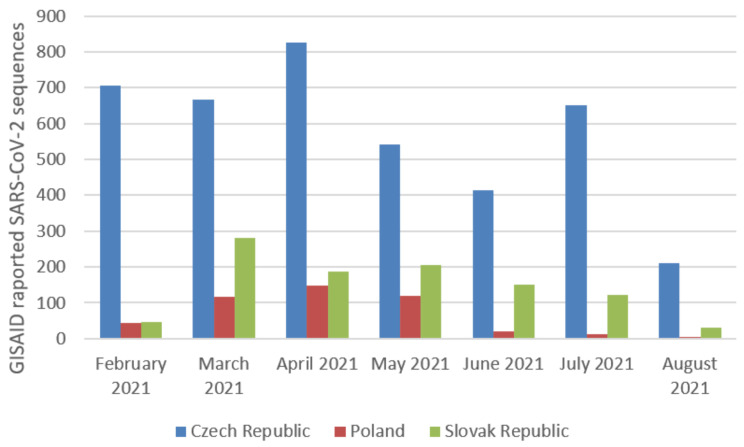
The number of reported SARS-CoV-2 sequences in GISAID for Poland (except Southern Poland), the Czech Republic, and Slovakia per 1 ml/n inhabitants. The largest number of sequenced samples per 1 million inhabitants was in the Czech Republic, followed by Slovakia. In Poland, the fewest samples were reported per 1 million inhabitants. The most samples were reported in the period from February to May 2021, which coincided with the third wave of the SARS-CoV-2 pandemic. During the holiday season, a decrease in the number of reported sequences can be seen.

**Figure 6:**
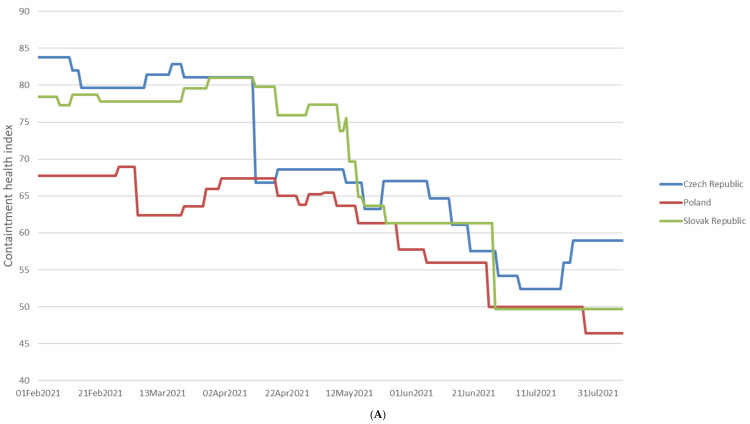

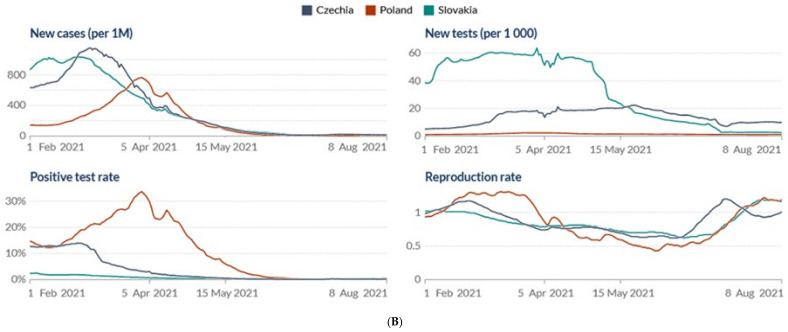
(**A**)**:** The containment health index for Poland, Slovakia, and the Czech Republic. The index is a composite measure based on thirteen policy response indicators including school closures, workplace closures, travel bans, testing policy, contact tracing, face coverings, and vaccine policy rescaled to a value from 0 to 100 (100 = strictest). If policies vary at the subnational level, the index is shown as the response level of the strictest subregion [10]. (**B**)**:** COVID-19 cases, tests, positive rate, and reproductive rate. Due to limited testing, the number of confirmed cases is lower than the true number of infections. Comparisons across countries are affected by the differences in testing politics and reporting methods [11].

The analysis of whole-genome sequences from Poland, the Czech Republic, and Slovakia was carried out based on the data available in GISAID. An analysis of 17,856 sequences reported in GISAID between February 2021 and 8th August 2021 in Poland was performed. The B.1.1.7 was the dominant variant among sequenced samples (92.57%). The other variants occurred with a much lower frequency (Figure 2). In the Czech Republic, 6825 samples were collected in the analyzed period. Similarly, the B.1.1.7 lineage dominated in the population with a frequency 65.89% (Figure 3). Out of 5596 reported samples in Slovakia, the frequency of variant B.1.1.7 was 80.21%. The other variants occurred with much lower frequencies (Figure 4). The numbers of GISAID SARS-CoV-2 reported sequences in Poland, the Czech Republic, and Slovakia per 1 ml/n inhabitants are presented in Figure 5. The Czech Republic reported the largest number of sequences per 1 million inhabitants followed by Slovakia. In Poland, the number of sequenced and reported samples was visibly smaller. Comparing the number of samples sequenced in Poland in the analyzed period (17,865) with the number of samples sequenced in Silesia (921), it can be seen that the research conducted in Southern Poland reflects the real picture of the pandemic in this region since the number of sequenced samples was large. Comparing Poland with the Czech Republic and Slovakia, the differences can also be seen in the containment health index, which is the lowest in Poland (Figure 6A). On the other hand, Figure 6B shows the statistics on new cases, tests, positive rate, and reproduction rate and also indicates that Poland’s response to the pandemic was the weakest.

### 2.3. The Phylogenetic Relationships of SARS-CoV-2 Clades

**Figure 7:**
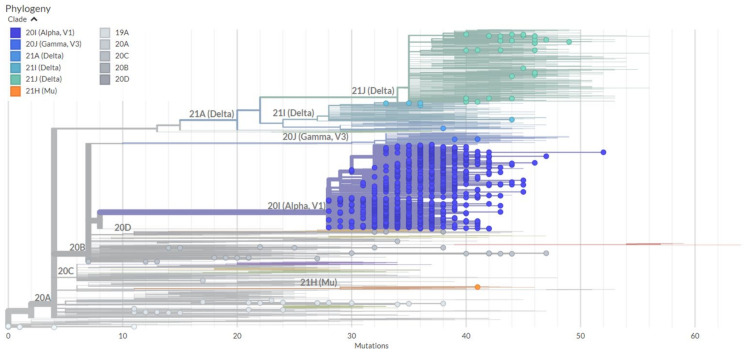
The maximum likelihood phylogeny of Southern Poland whole-genome sequences generated in Nextclade. The amino acid substitutions were used and the maximum likelihood phylogenetic tree was developed for each wave. The different shades of blue, green, gray, and orange dots in the phylogenetic tree show the distribution of SARS-CoV-2 samples in the respective clades. The lineages that share the same mutations group together. The longer lines mean more mutations and sequences on single long lines mean unique mutations. The B.1.617.2 clade carried the highest number of mutations, followed by B.1.1.7, while clade P.1 and B.1.621.1 showed different mutations. The B.1.621.1 clade, despite other clades’ spike protein mutations, also had the extra substitution Y145H, while P.1 had additional sequence changes resulting in R190S, T638I, T1027I, and V1176F. Legend: 20A (Pango: B.1.160), 20B (Pango: B.1.1.277), 20C (Pango: B.1.367), 20D (Pango: C.36), 20J (Pango: P.1.1), 20I (Pango: B.1.1.7), 21H (Pango: B.1.621), and 21A (Pango: B.1.617.2).

**Figure 8:**
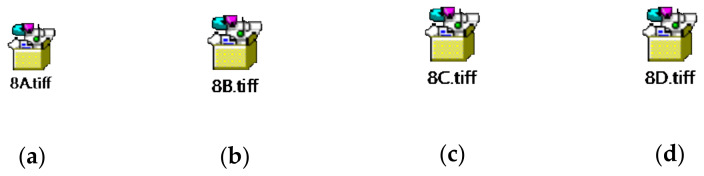
Maximum likelihood (ML) phylogeny of whole-genome sequences of analyzed populations. (**a**): Phylogeny of Silesia whole-genome sequences of SARS-CoV-2 (n = 814). The individual colors symbolize the variants and the red color represents the reference sequence. The PANGO nomenclature was used. (**b**): Phylogeny of Silesia whole-genome sequences of SARS-CoV-2 (n = 814) in the context of Poland as a whole (n = 17,865, except data presented in Figure 8). The individual colors symbolize the variants and the red color the reference sequence. Comparing Silesia with Poland, it can be seen that in both cases the dominant variant was lineage B.1.1.7. In Silesia, it performed with frequency of 79.37% (n = 730), while in the rest of Poland it accounted for 86.23% (n = 4654). In Poland, the common variants were B.1.221 (2.16%, n = 117) and B.1.258 (1.76%, n = 95), which were not found in Silesia. Additionally, in Poland there are variants from the AY lineage (2.67%, n = 144), present only in individual cases in Southern Poland (<1%). Moreover, variant B.1.617.2 (4.23%, n = 39) occurred with greater frequency in Southern Poland than in the other regions of the country (0.34%, n = 21). The PANGO nomenclature was used. (**c**): Phylogeny of Silesia whole-genome sequences of SARS-CoV-2 (n = 814) in the context of the Czech Republic (n = 6825) and Slovakia (n = 5596). The individual colors symbolize the variants and the red color the reference. The PANGO nomenclature was used. (**d**): Phylogeny of Silesia whole-genome sequences of SARS-CoV-2 (n = 814) in the context of the Czech Republic (n = 6825) and Slovakia (n = 5596). The individual colors symbolize countries. Comparing Silesia with the Czech Republic and Slovakia, it can be seen that the dominant variant was lineage B.1.1.7 in each case. In Silesia it performed with frequency of 79.37% (n = 730), while in the Czech Republic and in Slovakia it was present with frequencies of 61.04%, n = 738, and 74.02%, n = 704, respectively. Additionally, in Slovakia and the Czech Republic there were also variants from the AY lineage (27.87%, n = 337; 32.10%, n = 226), present only in individual cases in Southern Poland (<1%). Moreover, variant B.1.617.2 occurred with greater frequency in the Southern Poland (4.23%, n = 39) than in the Czech Republic (3.72%, n = 45) and Slovakia (1.7%, n = 12). The PANGO nomenclature was used.

The phylogenetic analysis of the tested samples from Southern Poland revealed the presence of two main clades: B.1.160 and B.1.153. The B.1.153 clade was derived from B.1.160, similar to B.1.367. The dominant lineage was B.1.1.7 derived from B.1.153. Variant B.1.617.2, which was a branch of B.1.160, replaced B.1.1.7 with time. Variant B.1.617.2 (S:A222V) had all the mutations of B.1.617.2, but, in addition, it carried a mutation in the spike protein at position A222V, which was confirmed in this study. Moreover, it also harbored additional amino acid mutations in ORF1a: ORF1a:P1640L, ORF1a:A3209V, ORF1a:V3718A, and ORF1a:T3750I and nucleotide substitutions at positions A5584G and C13019T [12]. Variant B.1.617.2 (N:G215C) is also a subpopulation of B.1.617.2. However, when analyzing the mutation in the spike protein, no additional mutations were noticed. Except for all B.1.617.2 variant mutations, it carried a mutation in the region encoding N protein N:G215C and substitutions at positions C8986T and A11332G. Moreover, it had extra mutations in the ORF region: ORF1a:A1306S, ORF1a:V2930L, ORF1a:T3255I, ORF1a:T3646A, ORF1b:A1918V, and ORF7b:T40I [12]. Moreover, clade B.1.621.1, which is a subpopulation of clade B.1.160, was observed. Moreover, a single case of clades B.1.1 and B.1.1.317 (not visible in Figure 7 but present in Figure 8A) was noted during the phylogenetic analysis. An illustration of phylogenetic relationships of SARS-CoV-2 clades from Southern Poland is presented in Figure 7, but due to the low transparency of the phylogeny generated in Nextclade, a phylogenetic tree construction in R v. 4.1.3 using libraries: ape, adegenet, phangorn, treeio, and ggtree was performed. The JC69 substitution model for the tree construction, and maximum likelihood method for tree building were used. An illustration of phylogenetic relationships of SARS-CoV-2 clades from Southern Poland is presented in Figure 8A. We also generated a phylogenetic tree for Silesia in the context of Poland as a whole (Figure 8B) and Poland divided into regions (Figure S2). A comparative analysis of Silesia with the Czech Republic and Slovakia was also carried out (Figure 8C,D). In each case, the dominant variant was B.1.1.7. In Silesia, however, we observed earlier appearance and faster spread of variant B.1.617.2 compared to Poland, the Czech Republic, and Slovakia, where AY (AY.122, AY.4, AY.42, AY.43, AY.5.5, AY.68, AY.9.2, and AY.98.1) lineages were more frequent. Due to the size of the files, the figures are presented in a PDF format.

### 2.4. Spike Protein Mutations Analysis

Among the 814 sequenced samples, a total of 180 spike mutations were identified. The most common mutation in the spike protein was substitution D614G which occurred in 805 samples (98.89%). Deletions ΔH69/V70 and ΔY144 were fixed in 740 (90.90%) and 722 (88.69%) sequences, respectively, while substitutions N501Y, P681H, A570D, D1118H, T716I, and S982A occurred with frequencies of 727 (89.31%), 726 (89.18%), 723 (88.82%), 722 (88.96%), 72 (88.45%), and 72 (88.45%), respectively. Other mutations were noticed with frequencies lower than 5%. The cross-correlation of the 20 most common mutations in the S protein occurring with a frequency of >2% is shown in Figure S1 in Supplementary Materials.

**Figure 9:**
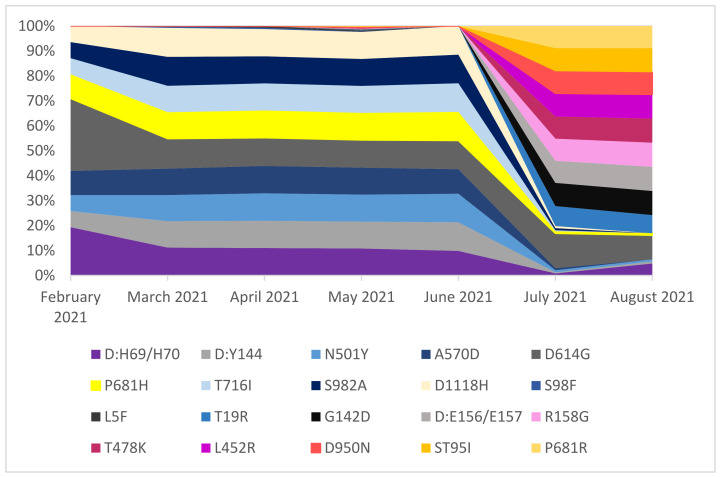
The percentage distribution of the major mutations in the SARS-CoV2 S protein circulating in Southern Poland from February 2021 to August 2021. Mutations present in more than 2% of the total sequenced samples are presented. The analysis was carried out in the Nextstrain domain.

The incidence of individual mutations strongly correlated with the currently circulating SARS-CoV-2 variant in Southern Poland. Along with the change of the dominant variant from B.1.1.7 to B.1.617.2 in July 2021, a change in the prevailing mutations was observed. In February 2021, when the B.1.160 clade dominated, the most common mutation was substitution D614G (100%). The other mutations occurred with varying frequencies, but not exceeding 90%. When the B.1.1.7 variant dominated the clades circulating in Southern Poland in March 2021, the number of mutations in the spike protein significantly increased. From March to June 2021, mutations ΔH69/V70, ΔY144, N501Y, A570D, D614G, P681H, T716I, S982A, and D1118H were present with a frequency of over 80%. In parallel with the introduction of the B.1.617.2 variant in July 2021, there was a sharp decrease in their incidence. However, other sequence changes correlated with the B.1.617.2 variant appearance: T19R, G142D, ΔE156/E157, R158G, L452M, L452R, D950N, ST95I, and P681R. The mutations common in both variants were: ΔH69/V70, ΔY144, N501Y, D614G, and P681H. However, these mutations were much less common among the B.1.617.2 variant [13]. The percentage distribution of the major mutations in the SARS-CoV-2 S protein from February 2021 to August 2021 is presented in Figure 9 and Table S1 in Supplementary Materials.

## 3. Discussion

SARS-CoV-2, which is responsible for the current pandemic, continuously evolves as genetic mutations occur during the replication of its genome. Whole-genome sequence analysis is strongly recommended to keep track of the circulating virus strains as new virus variants may cause increased infectivity and virulence, resistance to therapeutic agents, and immune evasion. The selection of specific SARS-CoV-2 variants related with reduced susceptibility to convalescent plasma was noticed during the course of a persistent infection in an immunocompromised host [14]. The persistent infection in an immunocompromised patient was pointed out by Choi et al. as a place of the accelerated viral evolution [15].

The analysis of 921 SARS-CoV-2 sequences collected within the timeframe from February 2021 to August 2021 in the Silesia region was performed to better the understanding of SARS-CoV-2 dissemination. Our research showed that VOCs established supremacy twice during this period, namely, variant B.1.1.7 became the most abundant in March 2021 and it was overtaken by variant B.1.617.2 in July 2021.

We analyzed the prevalence of SARS-CoV-2 lineages in the Silesia region in comparison with GISAID-reported data from the rest of Poland as well as the nearest neighboring countries: the Czech Republic and Slovakia. Regarding the dynamic of VOCs’ occurrence, we noticed that the B.1.1.7 lineage was introduced at the same time and spread with a similar pattern in all analyzed populations. In contrast, the B.1.617.2 lineage was found with a very high frequency in Silesia during the summer months, whereas it was identified with a frequency below 10% in the Czech Republic and Slovakia, where lineages AY.122, AY.43, and AY.4 dominated at that time. The earlier appearance of the B.1.617.2 variant in Silesia may be associated with the lifting of restrictions and the holiday period associated with travels. Since Silesia is a large agglomeration, with an airport, train station, and a large flow of travelers, the B.1.617.2 variant may have been imported from another region of the world.

Variant of concern (VOC) Alpha (lineage B.1.1.7) was first detected in southeast England in September 2020 and spread to become the dominant lineage in the United Kingdom in just a few months, and as of 29 March 2021, Alpha comprised roughly 95% of new SARS-CoV-2 infections in England [16]. In February 2021, the first month of our study, we observed variant B.1.1.7 as co-occurring with the B.1.258 and B.1.153 variants. In the period from March to June, it became the dominant lineage in our region. The prevalence of variant B.1.1.7 ended in July 2021, when it was displaced by B.1.617.2.

Serwin et al. analyzed SARS-CoV-2 variant evolution in Poland in the first year of the pandemic, namely from March 2020 to February 2021. The most frequently occurring variant in February 2021, the first month of our study, was the B.1.1.7 lineage, that was identified in 52% of variants [17]. At this time, in our Silesian cohort, 78% of sequences belonged to either the B.1.160 or B.1.153 variants, whereas the B.1.1.7 variant was just introduced but its prevalence increased rapidly up to 87% in March and 97% in April 2021. Interestingly, among samples collected in eastern Poland between the end of January and the first eleven days of February 2021, only 25% of sequences were classified as B.1.1.7, whereas B.1.1.74 was the most common variant [18]. GISAID data analysis of the dynamic of SARS-CoV-2 variants’ spread in the rest of Poland revealed an even more rapid increase in B.1.1.7 lineage. Namely, variant B.1.1.7 was identified in nearly 58% of variants in February 2021 and the peak frequency (98%) was attained in April, whereas B.1.258 and B.1.153 lineages dominant in Silesia were observed less frequently (7% and 2.6%, respectively). A similar dynamic of B.1.1.7 variant spread was noticed in Saxony-Anhalt, Germany, where it was observed for the first time in February 2021; then, the incidence of this variant increased tremendously up to 75% of all cases in March and nearly 100% in May 2021 [19]. The surveillance screening for lineage B.1.1.7 on 2 February 2021 performed in the Slovak Republic revealed its region-specific prevalence ranging from 52% to 85% in different provinces with an overall prevalence of 75%. A final screening on 3 March 2021 showed increased B.1.1.7 prevalence in all regions and an overall prevalence of 85% [20]. According to GISAID data, the number of B.1.1.7 variants increased from 69% in February to 98% in May with the most rapid increase between February to March. Similarly, in the Czech Republic the B.1.1.7 lineage appeared earlier and spread faster than in Silesia, as at the end of January–February it was the most abundantly represented lineage (>88%) [21]. However, only 79% of genomes reported from the Czech Republic in the GISAID database in February were qualified as B.1.1.7 lineage. Variant B.1.1.7 spread more rapidly also in Switzerland, where 95.9% of successfully sequenced genomes collected from 14 October 2020 to 28 February 2021 were assigned to this lineage, 3.9% were assigned to the B.1.351 lineage, and 0.2% to the P.1 lineage [20]. Thus, the above data show that in Silesia the B.1.1.7 variant was introduced later than in the neighboring countries; however, it displaced the earliest variants quickly, especially in the period between February and March 2021.

We can surmise that the differences in the dynamic of spread of the B.1.1.7 lineage observed between Poland and the neighboring countries may have arisen from the insufficient number of tests performed in Poland, which might have resulted in an underestimation of the rapidly increasing number of infected patients. The noticed time lag of B.1.1.7 spread in the Silesia area compared to the data from the rest of Poland may suggest that this variant reached other regions of the country earlier. This delay may be elucidated by various restrictions implemented by governments in individual districts depending on the number of newly diagnosed patients.

In late 2020, B.1.617 variants, including B.1.617.1 and B.1.617.2, were first detected in India and caused devastating epidemics before spreading globally [9,22,23]. The B.1.617.2 variant appeared among the Silesian population in May and subsequently it was detected in 67% of sequences collected in July and 92% of sequences in August. These observations diverged greatly from the situation in the other regions of Poland, Slovakia, and the Czech Republic. The analysis of whole-genome sequences reported to the GISAID database showed that July 2021 was the only month when B.1.617.2 variants were found in Poland with an incidence of 41%. In the Czech Republic, some cases of the B.1.617.2 variant were detected as early as March 2021 and then in the period from May to August; however, it was infrequent, as the maximum frequency of its occurrence (9%) was noticed in August. It should be mentioned that Silesia is one of the most densely populated regions in Poland, which facilitates virus spread and may in part be responsible for the observed inequalities in the dynamic of B.1.617.2 lineage incidence. In the UK, B.1.617.2 rapidly became the dominant circulating SARS-CoV-2 variant, from 0.09% at the beginning of April 2021 to >98% at the end of June 2021, displacing the B.1.1.7 variant which concomitantly decreased from 98% to 1.67% [24]. These observations are in accordance with data collected in the GISAID database where the greater increase in sequences belonging to the B.1.617.2 lineage was reported in July and August [25].

The inception of variants bearing ΔH69/V70, N439K, and D614G mutations took place before February 2020 [17] and in the first month of our study, it was the most abundant lineage (44%); then, it was dropping systematically and was replaced by the B.1.1.7 variant in June. This lineage was reported by Sewerin et al. as the dominant one in November and December 2020 and it was dominated by variant B.1.617.2 in January 2021 in Northern Poland [17]. Our analysis of the GISAID database revealed that the B.1.258 lineage was found in Poland only in February (7%). Similar results have been found in Slovakia, whereas in the Czech Republic 12–13% of sequences reported in February and March were classified as B.1.258, and in the further months only very few cases of this variant were noted. The rate of eradication of lineages circulating in the populations at the beginning of 2021 is correlated with the time of introduction and the rate of spreading of the B.1.1.7 lineage; therefore, because B.1.1.7 reached Silesia later than the rest of the areas of Poland, the rate of B.1.258 disappearing was slower.

It is interesting that variant B.1.213 introduced to Southern Poland in July was found neither in other regions of Poland nor in Europe during the whole period of our study. According to the GISAID database, the B.1.213 variant was observed in the Malopolska and Mazowsze regions, namely in May–June 2020 and in January 2021, respectively. Thus, the source of inflow of this lineage remains a mystery, as the previous one in Europe was found in Germany in April 2021. We cannot exclude that this lineage was circulating in the Polish population, although at the low level due to the presence of more efficient lineages, firstly B.1.17 and then B.1.617.2. Because of limited testing, it is likely that patients infected with a very rare lineage of SARS-CoV-2 were not detected.

In August 2021, we found in the Silesian population the B.621.1 lineage that was not observed either in Poland or in the countries nearby; however, it was reported from many European countries between April and October 2021. Therefore, the appearance of this variant may be the consequence of holiday travelling.

Basically, the SARS-CoV-2 variants found in the Silesia region were similar to that observed in the other regions of Poland as well as in the Czech Republic and Slovakia, where many Silesian residents work across the border and commute frequently. In all examined regions, a subsequent flip in the preponderance of variants in the period between February and August of 2021 was noticed; however, some differences in the time of introduction and the dynamic of spread of the lineages were spotted probably due to the differences in government pandemic policies reflected in the containment health index.

The number of missense mutations in the spike protein was relatively constant in analyzed sequences throughout the study, but we observed a significant change in the type of mutations over time related to changes to the currently circulating variants in Southern Poland. Most probably, the spectrum of the SARS-CoV-2 mutation is represented by many different viruses isolated from people around the world and their appearance in various places around the world is related to a convergent evolution influenced by the activity of viral RNA enzymes [26]. In February 2021, the B.1.160 was the dominant variant, followed by B.1.153. At that time, the B.1.1.7 clade accounted for only 22.22%, and still it was responsible for the greatest genetic variation. The samples identified as the B.1.1.7 variant carried a large number of the mutations (ΔY144, N501Y, A570D, P681H, T716I, S982A, and D1118H) during this period. The B.1 and B.1.153 clades carried only deletion ΔH69/H70 and substitution D614G. The analysis of mutations in the spike protein by Serwin et al. in the period from November 2020 to February 2021 in Northern Poland confirmed that the main sequence changes in the S protein before February 2021 were ΔH69/H70 and D614G [17]. Moreover, the authors mention that they excluded samples obtained before November 2020 from the mutation tests due to low genetic diversity, which shows that the increase in the molecular differentiation of the virus was associated with the second wave of the pandemic in Poland, in the fall of 2020. The D614G substitution occurred consistently with high frequency throughout Serwin’s study [17]. The increasing frequency of the D614G mutation suggests a selective advantage. Variants with the D614G mutation are associated with a higher viral load and affect younger patients, but there is no evidence that they cause higher mortality [27]. The authors observed an increase in the frequency of the mutations ΔH69/H70, ΔY144, P681H, T716I, S982A, A570D, N501Y, D1118H, and N439K over time. They noted that these were mutations associated with the B.1.1.7 variant. Interestingly, only four mutations, ΔH69/V70, P681H, S98F, and A222V, had significantly different frequencies from November 2020 to February 2021 in the case of clades other than B.1.1.7. The above observations suggest that in Southern Poland, as in Northern Poland, the dominant mutations before March 2021 were ΔH69/H70 and D614G. Analyzing February 2021 separately, as the only common month in both studies, it is seen that the dominant mutations in Poland were: ΔH69/H70 and D614G. In addition, in Southern Poland, the P681H substitution occurred with a frequency of 60%, while in Northern Poland it was present in only 33% of genomes. Correlations for missense mutations in the spike protein in Serwin et al. were also similar to those obtained in our work. In both studies, the coexistence of ΔH69/H70, ΔY144, P681H, T716I, S982A, A570D, N501Y, and D1118H was noted [17].

The above observations show that the spread of variant B.1.1.7 in March 2021 started a dynamic expansion of genetic variability, coinciding with the beginning of the third wave of the epidemic in Poland observed since March 2021, with the peak of new infections in early April and the peak of COVID-19-associated deaths in mid-April.

In July 2021, we noted that the B.1.1.7 variant was displaced by the B.1.617.2. The D614G mutation continued to occur with high frequency, while the frequency of deletion ΔH69/H70 decreased from 85.71% in June to 6.06% in July. The appearance of the B.1.617.2 clade was associated with completely new mutations in the spike protein, which first appeared in the study population together with B.1.617.2 in May 2021. The frequencies of mutations correlated with the B.1.1.7 variant dropped sharply in July 2021, and new mutations associated with the B.1.617.2 variant emerged: T19R, G142D, ΔE156/E157, R158G, T478K, L452R, D950N, ST95I, and P681R. The B.1.617.2 lineage was dominant during the fourth wave of the pandemic in Poland in autumn 2021.

Comparing our results with similar studies conducted around the world, slight differences in the missense mutations’ frequencies can be noticed. Ghosh et al. analyzed 77681 SARS-CoV-2 genomes collected from GISAID [28]. The study covered 98 countries over the period from January 2020 to July 2021. Comparing the February 2021 to July 2021 interval from the above study with our data, it can be concluded that the frequency of mutations in the S protein over time in Southern Poland coincides with the analysis of sequences from around the world. Both in Southern Poland and in the world, mutations related to the B.1.1.7 variant (ΔH69/H70, ΔY144, N501Y, A570D, P681H, T716I, S982A, and D1118H) occurred with a high frequency until June 2021 when they slowly gave way to mutations related to the B.1.617.2 variant (S98F, L5F, T19R, G142D, ΔE156/E157, R158G, T478K, L452R, D950N, ST95I, and P681R) in July 2021. Interestingly, the frequency of the D614G mutation calculated by Ghosh et al. has been decreasing since May 2020, while in Poland in the period from March 2020 to August 2021 it was the most common sequence change. Similarly, in our close neighborhood, in Germany, the D614G mutation occurred with a 100% frequency from February to May 2021, followed by the N501Y mutation [19]. Since the D614G substitution is derived from the B.1.160 variant, this mutation was quickly displaced in other regions of the world where the B.1.1.7 and B.1.617.2 variants predominated earlier. The remaining low-frequency mutations mentioned in the Ghosh et al. study were present in our research, also with low frequency. However, among the B.1.1.7 variant, there were S494P and SK1191 mutations, which we did not observe in Poland throughout the entire study, and E484K substitution which was noted only in three samples identified as P.1 clade [26]. The sequence changes E484K and K417N are responsible for increased viral transmission and decreased neutralization [29,30]. The A222V mutation is characteristic for 21I subclade, which at the beginning of dominance of the B.1.617.2 variant in our study occurred with a lower frequency.

When analyzing the above data, certain limitations should be taken into account. The number of new SARS-CoV-2 cases diagnosed in Poland did not reflect the pandemic situation, as compared to the Czech Republic and Slovakia, the percentage of positive tests was very high, and the total number of tests was well below accepted level. On the other hand, more samples in general were sequenced in Poland during the analyzed period. The pandemic management was less stringent in Poland, particularly in spring 2021, which could have resulted in increased new variant rapid introduction. Additionally, the number of sequenced and reported samples in GISAID per 1 million inhabitants was incomparably lower in Poland than in the Czech Republic and Slovakia. Therefore, it is possible that the differences in the obtained variant and mutation analysis result from the low number of tests performed in Poland and the lack of new cases reporting. It should also be noted that the number of samples sequenced in Silesia (921) during the analyzed period was large compared to rest of Poland (17,865), the Czech Republic (6825), and Slovakia (5596), which gives a fairly clear picture of the epidemic situation in Southern Poland from Alpha to Delta [31,32].

## 4. Materials and Methods

### 4.1. Study Group

Sequential analysis was performed on 921 samples, determined in RT-PCR tests (MediPAN 2G + FAST COVID Kit [Medicofarma, Radom, Poland], 2019-Novel-Coronavirus [2019-nCOV] Triplex RT-qPCR Detection Kit [Vazyme], Nanjing, China] MutaPLEX Coronavirus Real-Time-RT -PCR-Kit [Immunodiagnostic] and Xpert Xpress SARS-CoV-2 and Xpert Xpress SARS-CoV-2 / FLU / RSV [GeneXpert, Sunnyvale, CA, USA]) as positive, from February 2020 to August 2021. The number of samples tested in individual months was different. Study area covered Silesian Voivodeship in Southern Poland, with 4.5 million inhabitants and the highest population density in Poland and with the highest infection rate throughout the pandemic. Patients’ samples were collected in hospitals (671), hospital diagnostic points (119), other diagnostic mobile points (114). Samples and data for analysis came from 31 institutions/sources and were analyzed after prior anonymization. Probes with CT < 32 were qualified for the preparation of libraries.

### 4.2. SARS-CoV-2 Whole-Genome Sequencing (WGS)

RNA SARS-CoV-2 was isolated using Maxwell 48 RSC instrument (Promega, Fitchburg, WI, USA) and Maxwell RSC Viral TNA kit (Promega, Fitchburg, WI, USA). Qualitative and quantitative analysis of isolates was performed with Quantus fluorymeter (Promega) and Fragment Analyzer 5200 (Agilent). Reactions of RNA rewriting to cDNA or amplification conducted on the Biorad CFX96 Real-Time System (Biorad, Hercules, CA, USA) and Biorad C1000 Touch Thermal Cycler (Biorad, Hercules, CA, USA) devices. Sequencing was performed with two library preparation protocols: Respiratory Virus Oligo Panel RVOP (Illumina RNA Prep with Enrichment) for 873 samples and NEBNext ARTIC SARS-CoV-2 FS Library Prep Kit (NebNext, Ipswich, MA, USA) for 48 samples. Sequencing of the SARS-CoV-2 virus genome was performed on Illumina devices dedicated to NGS analyzes: miSeq and NextSeq 1000. After sequencing on an Illumina NGS system, data analysis proceeded using the DRAGEN pipelines: DRAGEN COVID Lineage (3.5.3 Version, https://www.illumina.com/products/by-type/informatics-products/basespace-sequence-hub/apps/dragen-covid-lineage.html, accessed on 14 June 2022, Pangolin: Max Ambiguous Rate 0.5) Pangolin: Max Ambiguous Rate 0.5) and DRAGEN RNA Pathogen Detection (3.5.16 Version, https://www.illumina.com/products/by-type/informatics-products/basespace-sequence-hub/apps/dragen-rna-pathogen-detection.html, K-mer Reference FASTA SARS-CoV-2_NC_045512.2, accessed on 14 June 2022, Reference Human Genome (hg38). Samples were qualitatively analyzed with coverage, mapped reads, duplicate reads, row reads, length, and % N.

### 4.3. Analysis of Sequencing Results

The sequencing results were analyzed, in which at least 95% of the sequence of the SARS-CoV-2 virus was identified. Clade assignment, mutation calling, and sequence quality checks were performed using the tools available on the website: https://clades.nextstrain.org/results (accessed on 27 January 2022). Phylogenetic trees were also generated using the Nextclade tools. The maximum likelihood phylogeny of Southern Poland whole-genome sequences generated in Nextclade was performed. The amino acid substitutions were used and the maximum likelihood phylogenetic tree was developed for each wave. The Nextstrain naming system for variants was used. All of the SARS-CoV-2 nucleotide sequences obtained in the study were deposited in GISAID with the EPI_ISL_2348500–EPI_ISL_2348510, EPI_ISL_2348617–EPI_ISL_2348621, EPI_ISL_2348648–EPI_ISL_2348650, EPI_ISL_2348654–EPI_ISL_2348656, EPI_ISL_2348659, EPI_ISL_2348665, EPI_ISL_2348667–EPI_ISL_2348729, EPI_ISL_2361502, EPI_ISL_2361521, EPI_ISL_2361522, EPI_ISL_2361618, EPI_ISL_2361691, EPI_ISL_2361692, EPI_ISL_2361712, EPI_ISL_2361743–EPI_ISL_2361745, EPI_ISL_2362041–EPI_ISL_2362044, EPI_ISL_2402633–EPI_ISL_2402810, EPI_ISL_2616369–EPI_ISL_2616521, EPI_ISL_2886201–EPI_ISL_2886213, EPI_ISL_2886433, EPI_ISL_2886434, EPI_ISL_2886436, EPI_ISL_4030118–EPI_ISL_4030278, EPI_ISL_4051105–EPI_ISL_4051599, EPI_ISL_4051617–EPI_ISL_4051643, and EPI_ISL_5315111–EPI_ISL_5315145.

### 4.4. Subsampling of the Data

For the subsampling of the SARS-CoV-2 sequences, we utilized a qiime2 genome-sampler workflow. Firstly, we imported the FASTA data and tsv metadata with qiime tools imported for both the context sequences and focal sequences (the sequences presented in this paper). We filtered the sequences to keep only those which had metadata. Furthermore, we filtered the datasets with qiime genome-sampler filter-seqs to retain sequences with at most 0.01 proportion of ambiguous bases. Next, we performed the data subsampling with the qiime genome-sampler sample–longitudinal with respect to the date, and qiime genome-sampler sample–diversity with respect to cluster sequences with a percent identity threshold of 99.95%, and selected the centroid sequence from each cluster to include in downstream analyses. Lastly, we sampled the data near neighbors of the focal sequences. We selected 3 sequences at random of the near-neighbor sequences, where the location metadata had equal probability of being selected for an inclusion in the downstream analysis.

Following this, we added to the dataset a reference SARS-CoV-2 sequence and performed a multialignment of the FASTA sequences with mafft software. To avoid spurious results, we applied a mask filter to positions that are likely uninformative according to the file shared by the Arctic Network Consortium [33].

### 4.5. Phylogenetic Tree Construction

We performed a phylogenetic tree construction in R v. 4.1.3 (https://cran.r-project.org/bin/windows/base/old/4.1.3/, accessed on 16 May 2022) using libraries: ape, adegenet, phangorn, treeio, and ggtree. We used a JC69 substitution model for the tree construction, and maximum likelihood method for tree building. We removed sites where ‘N’ bases remained.

### 4.6. Statistics

The Spearman test was used to determine the correlation between major mutations in the S protein and between individual mutations and the main clades circulating in Southern Poland. For all tests, *p* ≤ 0.05 was considered as statistically significant. Data were analyzed by use of STATISTICA v. 13.0 software (StatSoft, Cracow, Polska) on Windows platform (Microsoft Corp., Redmond, DC, USA).

## 5. Conclusions

The epidemiological situation with the SARS-CoV-2 lineages is variable, and the new VOCs’ emergence in the various regions of the world is highly probable. The European Center for Disease Prevention and Control (ECDC) and the World Health Organization (WHO) strongly recommend undertaking efforts to identify the circulating viral lineages in order to monitor the distribution of VOCs, using whole-genome sequencing for surveillance. In Silesia, a region of very high population density, we performed a sequencing-based surveillance program to track the introduction and spreading of SARS-CoV-2 lineages, which is critical to gain knowledge on the transmission of new variants. The accumulation of single or multiple mutations in the SARS-CoV-2 genome can lead to the emergence of more pathogenic variants; thus, it is crucial to understand the new variants’ dynamics and to assess their impact on the rate of transmission, susceptibility to immunological response, and therapy. Our results provide a comprehensive view of mutation and lineage identification of representative SARS-CoV-2 samples collected between February and August 2021 from the Silesia region. Our data provide evidence of the changing frequency of dominant variants from B.1.258 and B.1.153, predominant in February, through to the most frequent from March until July, the B.1.1.7 variant, to B.1.617.2 in August. The data reported in this study showed that in Southern Poland, the introduction of many lineages took place at a similar time as in Poland, the Czech Republic, and Slovakia; however, some differences in the dynamic of its spreading were noticed. The effective and timely genome surveillance of viral sequences is worthwhile for effective prevention and control.

## Data Availability

All of the SARS-CoV-2 nucleotide sequences obtained in the study have been deposited in GISAID with the EPI_ISL_2348500–EPI_ISL_2348510, EPI_ISL_2348617–EPI_ISL_2348621, EPI_ISL_2348648–EPI_ISL_2348650, EPI_ISL_2348654–EPI_ISL_2348656, EPI_ISL_2348659, EPI_ISL_2348665, EPI_ISL_2348667–EPI_ISL_2348729, EPI_ISL_2361502, EPI_ISL_2361521, EPI_ISL_2361522, EPI_ISL_2361618, EPI_ISL_2361691, EPI_ISL_2361692, EPI_ISL_2361712, EPI_ISL_2361743–EPI_ISL_2361745, EPI_ISL_2362041–EPI_ISL_2362044, EPI_ISL_2402633–EPI_ISL_2402810, EPI_ISL_2616369–EPI_ISL_2616521, EPI_ISL_2886201–EPI_ISL_2886213, EPI_ISL_2886433, EPI_ISL_2886434, EPI_ISL_2886436, EPI_ISL_4030118–EPI_ISL_4030278, EPI_ISL_4051105–EPI_ISL_4051599, EPI_ISL_4051617–EPI_ISL_4051643, and EPI_ISL_5315111–EPI_ISL_5315145 accession numbers.

## Supplementary Materials

The following supporting information can be downloaded at: https://www.mdpi.com/article/10.3390/pathogens11070780/s1, Figure S1: Correlation plot of major S protein mutations found in sequenced samples; Figure S2: Maximum likelihood (ML) phylogeny of Silesia whole-genome sequences of SARS-CoV-2 (n = 814) in the context of Poland divided into regions; Table S1: The frequency of mutations in the spike protein over time [%].
