# Supplementary material for: From Alpha to Delta—Genetic Epidemiology of SARS-CoV-2 (hCoV-19) in Southern Poland
Source: Pathogens. 2022 Jul 8;11(7):780. doi: 10.3390/pathogens11070780 (PMC9316897; doi:10.3390/pathogens11070780)
Supplement: Supplementary file 1 [file pathogens-11-00780-s001.zip › Figure S1.pdf]

| | ΔY144 | N501Y | A570D | D614G | P681H | T716I | S982A | D1118H | G142D | R158G | D950N | ΔE156/ E157 | L452R | P681R | L452M | L5F | T19R | S98F | ST95I |
|---|---|---|---|---|---|---|---|---|---|---|---|---|---|---|---|---|---|---|---|
| ΔH69/V70 | 0.88 | 0.86 | 0.89 | 0.28 | 0.86 | 0.87 | 0.87 | 0.89 | -0.68 | -0.66 | -0.68 | -0.67 | -0.67 | -0.66 | -0.64 | 0.04 | -0.62 | 0.01 | -0.03 |
| ΔY144 | | 0.95 | 0.96 | 0.26 | 0.95 | 0.95 | 0.95 | 0.96 | -0.63 | -0.61 | -0.61 | -0.62 | -0.62 | -0.62 | -0.59 | 0.05 | -0.58 | 0.01 | -0.26 |
| N501Y | | | 0.96 | 0.27 | 0.94 | 0.94 | 0.94 | 0.96 | -0.65 | -0.63 | -0.63 | -0.64 | -0.64 | -0.63 | -0.61 | 0.05 | -0.59 | 0.01 | -0.28 |
| A570D | | | | 0.26 | 0.96 | 0.97 | 0.97 | 0.98 | -0.63 | -0.61 | -0.63 | -0.62 | -0.60 | -0.62 | -0.60 | 0.05 | -0.58 | 0.01 | -0.30 |
| D614G | | | | | 0.30 | 0.25 | 0.25 | -0.03 | -0.03 | -0.03 | -0.03 | -0.03 | -0.03 | -0.03 | -0.09 | -0.03 | -0.03 | -0.03 | 0.02 |
| P681H | | | | | | 0.95 | 0.93 | 0.95 | -0.64 | -0.63 | -0.63 | -0.63 | -0.64 | -0.63 | -0.63 | 0.03 | -0.59 | 0.1 | -0.28 |
| T716I | | | | | | | 0.96 | 0.98 | -0.62 | -0.61 | -0.62 | -0.61 | -0.61 | -0.60 | -0.59 | 0.02 | -0.57 | 0.01 | -0.28 |
| S982A | | | | | | | | 0.99 | -0.62 | -0.60 | -0.62 | -0.61 | -0.61 | -0.60 | -0.59 | 0.05 | -0.57 | 0.01 | -0.29 |
| D1118H | | | | | | | | | -0.63 | -0.61 | -0.63 | -0.62 | -0.62 | -0.61 | -0.59 | 0.05 | -0.57 | 0.01 | -0.29 |
| G142D | | | | | | | | | | 0.97 | 0.97 | 0.99 | 0.99 | 0.97 | 0.92 | -0.05 | 0.92 | -0.008 | 0.45 |
| R158G | | | | | | | | | | | 0.94 | 0.99 | 0.96 | 0.95 | 0.89 | -0.05 | 0.98 | -0.007 | 0.41 |
| D950N | | | | | | | | | | | | 0.96 | 0.99 | 0.95 | 0.92 | -0.05 | 0.89 | -0.007 | 0.45 |
| ΔE156/ E157 | | | | | | | | | | | | | 0.97 | 0.96 | 0.90 | -0.04 | 0.90 | -0.007 | 0.42 |
| L452R | | | | | | | | | | | | | | 0.96 | 0.93 | -0.05 | 0.90 | -0.007 | 0.42 |
| P681R | | | | | | | | | | | | | | | 0.88 | -0.04 | 0.88 | -0.007 | 0.42 |
| T478K | | | | | | | | | | | | | | | | -0.04 | 0.82 | -0.007 | 0.40 |
| L5F | | | | | | | | | | | | | | | | | -0.04 | -0.007 | -0.03 |
| T19R | | | | | | | | | | | | | | | | | | -0.007 | 0.45 |
| S98F | | | | | | | | | | | | | | | | | | | -0.3 |

Positive corelations

Negative corelations

**Figure S1.** Correlation plot of major S protein mutations found in sequenced samples. Positive correlations are marked in red and negative ones in blue. The intensity of the color is directly proportional to the strength of the correlation. The Spearman test was used to assess the correlation, and the level of statistical significance was p <0.05. Mutations characteristic for the B.1.1.7 lineage strongly correlate with each other, while remaining in a negative correlation with mutations occurring among the variant B.1.617.2 . Similarly, sequence changes occurring in the B.1.617.2 variant strongly correlate with each other.
