# Supplementary material for: From Alpha to Delta—Genetic Epidemiology of SARS-CoV-2 (hCoV-19) in Southern Poland
Source: Pathogens. 2022 Jul 8;11(7):780. doi: 10.3390/pathogens11070780 (PMC9316897; doi:10.3390/pathogens11070780)
Supplement: Supplementary file 1 [file pathogens-11-00780-s001.zip › Table S1.pdf]

Table S1. The frequency of mutations in the spike protein over time [%]. The analysis was carried out in the Nextstrain domain.

Mutation	February 2021	March 2021	April 2021	May 2021	June 2021	July 2021	August 2021
ΔH69/V70 (-)	66.66	91.59	96.39	96.66	85.71	6.06	46.15
ΔY144	22.22	86.55	95.79	96.33	100	3.03	7.69
N501Y	22.22	85.71	96.99	96.66	100	6.06	7.69
A570D	33.33	86.55	96.09	96.66	85.71	6.06	0
D614G	100	98.31	99.09	99	100	100	92.3
P681H	33.33	86.55	96.09	96.66	100	6.06	7.69
T716I	22.22	86.55	95.19	96.66	100	3.03	0
S982A	22.22	95.19	95.19	96.66	100	6.06	0
D1118H	22.22	95.19	95.79	96.66	100	6.06	0
S98F	0	4.20	4.50	1	0	0	0
L5F	0	1.68	5.70	4	0	0	0
T19R	0	0	0	1.66	0	57.57	69.23
G142D	0	0	0	1.66	0	66.66	92.3
ΔE156/E157 (-)	0	0	0	1.66	0	63.63	92.3
R158G	0	0	0.3	1.66	0	63.63	92.3
T478K	0	0	0.3	3	0	63.63	92.3
L452R	0	0	0	1.66	0	66.66	92.3
D950N	0	0	0	1.66	0	63.63	84.61
ST95I	0	0	0	1.66	0	66.66	92.3
P681R	0	0	0	1.66	0	63.63	84.61
